# Temporary Relocation during Rest Periods: Relocation Stress and Other Factors Influence Hair Cortisol Concentrations in Horses

**DOI:** 10.3390/ani10040642

**Published:** 2020-04-08

**Authors:** Jaume Gardela, Annaïs Carbajal, Oriol Tallo-Parra, Sergi Olvera-Maneu, Manuel Álvarez-Rodríguez, Eduard Jose-Cunilleras, Manel López-Béjar

**Affiliations:** 1 Department of Animal Health and Anatomy, Veterinary Faculty, Universitat Autònoma de Barcelona, Bellaterra (Cerdanyola del Vallès), 08193 Barcelona, Spain; jaume.gardela@uab.cat (J.G.); anais.carbajal@uab.cat (A.C.); sergi.olvera@uab.cat (S.O.-M.); manuel.alvarez.rodriguez@uab.cat (M.Á.-R.); 2 Department of Animal and Food Sciences, Veterinary Faculty, Universitat Autònoma de Barcelona, Bellaterra (Cerdanyola del Vallès), 08193 Barcelona, Spain; oriol.tallo@uab.cat; 3 Department of Biomedical and Clinical Sciences (BKV), Division of Children’s and Women Health (BKH), Obstetrics and Gynecology, Linköping University, Linköping, 58183 Östergötland, Sweden; 4 Department of Animal Medicine and Surgery, Universitat Autònoma de Barcelona, Bellaterra (Cerdanyola del Vallès), 08193 Barcelona, Spain; eduard.jose.cunilleras@uab.cat; 5 College of Veterinary Medicine, Western University of Health Sciences, 309 East Second Street, Pomona, CA 91766, USA

**Keywords:** hair cortisol, horse, stress, relocation, rest period, welfare

## Abstract

**Simple Summary:**

Horses are frequently transported and exposed to a new environment for sport competition or working tasks and must readapt to their original conditions after a temporary relocation. The objective of this study was to determine if a temporary relocation, and multiple factors associated with a rest period, affect the adrenal response through the analysis of hair cortisol concentrations (HCCs) in horses. Cortisol is a glucocorticoid released after the activation of the hypothalamic–pituitary–adrenal axis and its assessment is being increasingly used as a bioindicator of stress response. Results showed that changes in the daily routine of the animals, including a supposed rest period, increased the HCCs. However, the risk of using low statistical power due to the small sample size cannot be completely ruled out. The elevation in HCCs could be a consequence of the change in the horses’ environmental and routine conditions, which could, in turn, have an impact on their welfare.

**Abstract:**

Horse transportation for temporary relocation during rest periods is a common and widespread practice among horse owners, either from sport competition or working tasks. This study aimed to determine the effect of a relocation period and the multiple factors associated with a rest period on hair cortisol concentrations (HCCs) in horses. Additionally, this study reports the seasonal effect on HCCs and hair growth over a year. Thirteen police horses, Pure Spanish stallions of various ages (5–13 y), were selected to participate in this study. Hair sample collection was carried out approximately every 30 d for seven months (Study 1) and a year (Study 2). Cortisol determinations were performed by enzyme immunoassay. Interestingly, Study 1 revealed that relocated horses (*n* = 4) exhibited elevated HCCs compared with control horses (*n* = 4) after the relocation period (*p* < 0.05). Study 2 (*n* = 5) showed higher HCCs during summer compared with autumn and winter, and higher hair growth rates in winter compared with the other seasons (*p* < 0.05). Relocated horses had higher HCCs, suggesting a change in their welfare status, probably related to the sudden change in their surrounding conditions. However, these results should be interpreted cautiously due to the low sample size used. The nature of the relationship between HCCs and horse welfare needs to be further examined.

## 1. Introduction

Even though machine power has replaced animal force for transport and traction, horses are still used as working animals (e.g., police or carriage horses). Horses are also used in competitive sports like dressage, endurance riding, show jumping, and other disciplines. In the same way that human professional athletes interrupt their competition period, horses are subjected to a compulsory rest period from the competition in endurance disciplines [1]. Nonetheless, the word “rest” is a misnomer as horses continue to train to maintain their athletic performance [2,3]. For logistical reasons, horse owners usually relocate their animals during the rest periods. To the authors’ knowledge, no studies have been performed to assess the effects of a temporary relocation and rest period on horses’ welfare. 

Like all mammals, horses are often recurrently exposed to different kinds of stressors and commonly adapt favorably to them. For instance, transport and other stressful situations increase the hypothalamic–pituitary–adrenal (HPA) axis activity releasing cortisol, which produces changes in behavior and energy balance to facilitate coping with the stressor [4]. Because salivary cortisol concentrations correlate well with cortisol concentrations in plasma in horses [5], cortisol in saliva reliably mirrors changes as a result of HPA axis activation. Other matrixes, like feces and urine, have also been used for cortisol determination [6,7,8]. Plasma, salivary, fecal, and urine cortisol concentrations reflect acute stressors, but they are not able to represent a long-term retrospective integrative stress response, as hair cortisol does [9]. 

Hair cortisol allows reliable monitoring of the accumulation of cortisol during the hair growth cycle [9,10,11]. Because of the lipophilic nature of steroid hormones, these compounds penetrate the hair shaft via passive diffusion from blood during the hair growth cycle [11,12]. In order to obtain a sample with growing hairs, the “shave–reshave” method is frequently used [9]. The method consists on shaving a certain area at the beginning of the study, discarding this hair sample from the analysis. After a period of interest, the regrown hair is reshaved [11,13]. The hair obtained is therefore, representative of the cortisol accumulation and HPA axis activity between the two-time points of interest. However, diverse hair-specific characteristics such as hair growth cycle, the growth rate of the sampling region, seasonal shedding rhythm, and hair color can be a source of variation and should be considered when using hair cortisol concentrations (HCCs) as a presumptive stress indicator [9].

Hair cortisol concentrations can be used as a tool to assess chronic stress or long-term activity of the HPA but not for occasional and sporadic stress events [14]. Experimental repeated stimulations with adrenocorticotropic hormone (ACTH) increased HCCs [15,16,17,18], but single doses of ACTH were insufficient to affect HCCs [14,19], indicating the robustness of HCCs against sporadic stress responses. Additionally, repeated stimulations with corticotropin-releasing hormone (CRH) increased the HCCs in CRH-treated animals compared with non-treated animals [20]. Jointly, the reports suggest that an accumulation of hair cortisol reflects repeated or chronic stimulation of the HPA axis.

Recent studies have described the long-term HPA axis activity in horses through the analysis of HCCs [21,22,23]. The assessment of the HPA axis activity has become a common approach to study stress and animal welfare [4] along with measurements of other end-points of the stress response [24].

This study aimed to determine if HCCs in horses could be affected by relocation stress and the multiple factors associated with a rest period. For this purpose, the long-term HPA axis response in a group of horses from the mounted police was evaluated using HCCs during a temporary relocation and rest period. Additionally, we assessed if other factors affect HCCs, such as endogenous factors (age, hair color, and health status), seasonality, and the hair growth rate.

## 2. Materials and Methods 

Horses were managed following the principles and guidelines of the Ethics Committee on Animal and Human Experimentation from the Universitat Autònoma de Barcelona. Informed consent from the staff of the mounted unit of the Municipal Police of Barcelona and the veterinary staff responsible of the care of the animals was obtained prior to the initiation of the study. No other manipulation different to shaving a small area of the abdomen was performed.

### 2.1. Hair Sampling and Storage

Hair was collected by shaving a 10 × 17 cm area from the ventral abdomen, left of mid-line and caudal to the sternum. Hair was collected at the skin level using single-use razors for each horse to avoid cross-contamination. Care was taken to avoid skin damage. At each sampling, the entire area was shaved again. About 0.3 to 0.4 g of hair were collected per animal and sample. Samples were stored in individually identified envelopes kept in the dark at room temperature until analytical processing.

### 2.2. Hair Steroid Extraction

Steroid extraction was performed following a methanol-based protocol previously validated for hair samples [25]. Briefly, 250 ± 3 mg of hair from each sample were weighed with a precision scale and placed in 15 mL conical tubes. Three washes were performed with 2.5 mL of isopropanol (2-propanol 99.5%, Scharlab S.L., Sentmenat, Spain) and mixed with a vortex mixer for 2.5 min each. The supernatant of each wash was removed by decantation. After washing, samples were dried for 36 h at room temperature. Once dried, the hair sample was powdered using a ball mill for 4 min at 25 Hz (MM200, Retsch, Haan, Germany; 10 mL stainless-steel grinding jars; two 12 mm stainless-steel grinding balls). A total of 50 ± 0.5 mg of powdered hair was weighed and placed in 2 mL Eppendorf tubes with 1.5 mL of pure methanol. Incubation was performed for 18 h at 30 °C under moderate shaking (G24 Environmental Incubator Shaker, New Brunswick Scientific CO Inc., Edison, NJ, USA). After incubation, samples were centrifuged at 7000× *g* for 2 min at 25 °C (Z300K Refrigerated Bench Top Centrifuge, Hermle Labortechnik GmbH, Wehingen, Germany). Then, 0.75 mL of supernatant was transferred to a 1.5 mL Eppendorf tube and placed open in an oven (Heraeus model T6; Kendro® Laboratory Products, Langenselbold, Germany) at 38 °C until complete evaporation. Once evaporated, 0.2 mL of buffer solution included in the enzyme immunoassay (EIA) kit (Cortisol ELISA KIT; Neogen Corporation, Ayr, UK) was added to each 1.5 mL Eppendorf tube and vortexed for 30–60 s to reconstitute the dried extracts. Immediately after reconstitution, hormone extracts were stored at −20 °C until their determination.

### 2.3. Hair Cortisol Detection and Validation Tests

Hair cortisol concentrations were determined using cortisol EIA detection kits (Cortisol ELISA KIT; Neogen Corporation, Ayr, UK) with a sensitivity of 0.32 pg cortisol/mg of hair. The precision within the test was assessed by calculating intra-assay coefficients of variation (CV, where CV = SD/mean × 100) from all duplicate or triplicate samples analyzed. The inter-assay coefficients of variation were calculated from pool samples with markedly different concentrations and analyzed per duplicate in each EIA kit. Linearity under dilution assesses specificity and accuracy, and was calculated by diluting the pool sample at 1:2, 1:5 and 1:8 ratios with the buffer solution included in the EIA kit. The spike-and-recovery test assesses accuracy and was calculated by adding to 50, 100 and 200 μL of pool sample to 200, 100 and 50 μL of pure standard cortisol solution, respectively. Combinations were repeated with three different pure standard cortisol solutions (20, 2, and 0.2 ng/mL) from the initial solution included in the EIA kit. According to the manufacturer, cross-reactivity of the EIA antibody with other steroids is as follows: prednisolone 47.4%, cortisone 15.7%, 11-deoxycortisol 15.0%, prednisone 7.83%, corticosterone 4.81%, 6β-hydroxycortisol 1.37%, 17-hydroxyprogesterone 1.36%, deoxycorticosterone 0.94%. Steroids with a cross-reactivity <0.06% are not presented.

### 2.4. Study 1: Relocation Effect on HCCs

A total of 8 Pure Spanish stallions of the Municipal Police of Barcelona, Spain, aged between 5 and 13 y (8.7 ± 2.6 y on average ±SD), were included in this study. The body condition score (BCS) was evaluated using the 0 (emaciated) to 5 (extremely fat) BCS scale [26]. For all the animals included in the study, BCS was 3 (moderate good body condition). Four horses on duty during the entire study period were used as the control group (2 bay and 2 gray), whereas four individuals (relocated horses; 2 bay and 2 gray) were temporally relocated during a specified period in the summer of 2016. 

Hair samples (*n* = 56) were collected approximately every 30 d (33.7 ± 7.1 d, mean ±SD) between August 2016 and February 2017 by shaving the same anatomical area. The hair collected at each sampling was only the new hair grown after the previous collection. 

During on-duty periods, all horses were housed in the same building, in indoor individual conventional stalls (2 × 2 m) with wood-chip bedding, with ad libitum access to water, and fed eight times a day (combination of forage, pelleted ration, bran, and fresh grass). Generally, horses trained daily either on the treadmill, patrolling peripheral areas of the city, or performing exercises in the outdoor arena. Transportation to peripheral areas lasted approximately 20–25 min/trip.

Relocated horses were driven to a new location 39.5 km away and 45 min drive in a horse truck from the main stables for a rest period of 22 d (12–31 August). During this period, horses were housed individually in outdoor paddocks (3 × 4 m) with ad libitum access to water, fed three times a day (combination of forage and pelleted ration), with no routine work or training, unknown stable staff, and visual and olfactory contact with unknown male and female horses. After this period, the horses were returned to the main stables of the municipal police under the same conditions as during on-duty periods.

The vehicle used for transportation in both situations was a commercial two-horse van, and each animal was loaded into the truck without the use of force and within 5 min. 

Clinical examination findings were recorded routinely by the veterinary staff. Horses’ health status was classified depending on the absence (healthy) or presence (non-healthy) of at least one clinical condition or disease in the clinical history during the 30 d before the hair sampling. Horses with the presence of at least one clinical condition or disease during the hair growth period of a sample were classified as non-healthy for that month. Clinical conditions and disease status included wound/abrasion (4 episodes affecting 3 horses), coronitis (3 episodes affecting 1 horse), lameness (1 episode affecting 1 horse) and back pain (1 episode affecting 1 horse).

### 2.5. Study 2: Seasonal Effect on HCCs and Hair Growth

Five Pure Spanish stallions of the Municipal Police of Barcelona, Spain, aged between 7 and 13 y (8.8 ± 2.7 y on average ±SD), were included in this study. Their BCS was 3 (moderate good body condition). Hair samples (*n* = 53) were collected approximately every 30 d (34.4 ± 3.6 d, mean ±SD) between November 2016 and October 2017 by shaving the same anatomical area. The hair collected on each occasion was only the hair that had grown since the previous collection. Additionally, the length of 25 randomly selected hair samples was measured with precision calipers (±0.05 mm; Metric, Spain).

All horses were housed in the same building, in indoor individual conventional stalls (2 × 2 m) with wood-chip bedding, with ad libitum access to water, and fed eight times a day (combination of forage, pelleted ration, bran, and fresh grass). The routine work of horses was the same as the daily training of Study 1 horses. Transportation to peripheral areas lasted approximately 20–25 min/trip.

Hair samplings were classified per month and according to the season as spring, April to June; summer, July to September; autumn, October to December; and winter, January to March.

### 2.6. Statistical Analyses

Statistical analyses were performed using GraphPad software (version 8.0.2) and RStudio software (R version 3.4.4). All data were checked for outliers and normal distribution using graphic tests (QQ-plot and box-plot) and the numeric test Shapiro–Wilk. The hair cortisol concentration data were not normally distributed. Transformed data restored normality using the inverse distribution (1/x). The significance level for all data was set at *p* < 0.05. Data are presented as median (25% percentile, 75% percentile) unless otherwise stated.

Cortisol biochemical validations were analyzed using Pearson’s product moment correlation to evaluate the correlation between obtained and expected values from serial dilutions and spiked pool extracts with cortisol standard. 

Study 1 data were analyzed using a repeated measures ANOVA. Post hoc comparisons over time and groups were performed using the Sidak’s multiple comparisons test. An additional analysis was performed using a linear mixed model, including age, hair color, and health status as dependent factors and individuals as independent factors. Post hoc comparisons over time were performed using Tukey’s multiple comparisons test. 

Study 2 data were analyzed using a mixed-effects model to analyze HCCs among months and a one-way ANOVA to analyze HCCs among seasons. Post hoc comparisons over time were performed using Tukey’s multiple comparisons test. Hair growth was analyzed by mixed-effects model. Post hoc comparisons over time were performed using Tukey’s multiple comparisons test. 

## 3. Results

### 3.1. Biochemical Validation of the Enzyme Immunoassay

Intra-assay variation computed for the mean of 11 replicate tests ranged from 2.07 to 0.07 ng cortisol/mL. Intra-assay and inter-assay CV were 6.52% and 6.82%, respectively. The linearity under dilution study provided R^2^ of 0.99 (*p* < 0.001) and the spike-and-recovery test provided R^2^ of 0.869 (*p* = 0.005). Replicates used in the spike-and-recovery test ranged from 2.07 to 0.62 ng cortisol/mL. The average recovery percentage from the spike-and-recovery test was 116.23 ± 25.15%.

### 3.2. Study 1: Relocation Effect on HCCs

**Figure 1:**
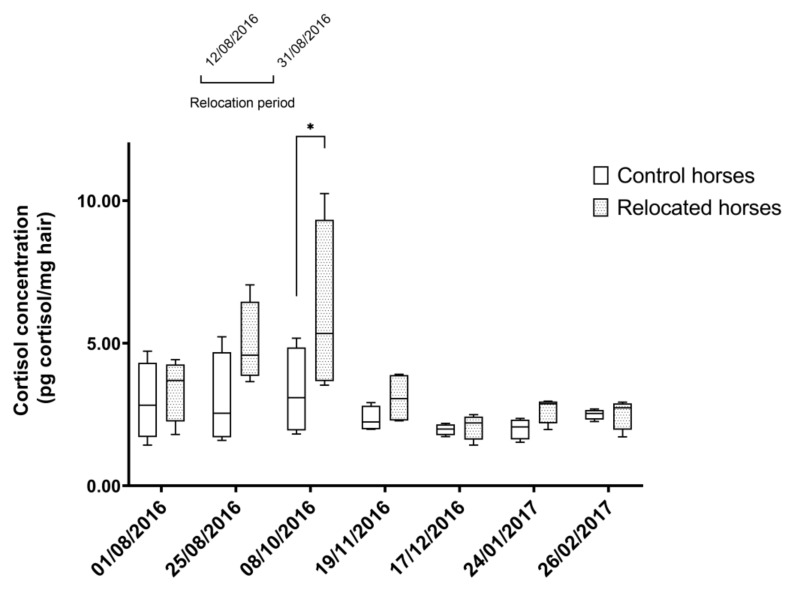
Hair cortisol concentrations in control and relocated horses during the study period. Hair cortisol concentrations (HCCs) (pg cortisol/mg hair) in control horses (N = 4, n = 28) and HCCs (pg cortisol/mg hair) in relocated horses (N = 4, n = 28) over the time of the study. Relocation period took place between 12th of August 2016 and 31st of August 2016. Asterisks represent significant differences between groups (*p* < 0.05). Median (25% percentile, 75% percentile). N = number of horses per group; n = number of determinations of HCCs.

Relocated horses and control horses had similar HCCs during the study (*p* > 0.9) except for the third sampling point (October 2016), when relocated horses had higher HCCs compared to control horses (5.34 (3.67, 9.33) and 3.09 (1.94, 4.85), respectively; *p* = 0.02), approximately a month after the end of the relocation period (Figure 1). The linear mixed model confirmed no effect of age, hair color (bay vs. gray), and health status on HCCs (*p* > 0.05). 

### 3.3. Study 2: Seasonal Effect on HCCs and Hair Growth

**Figure 2:**
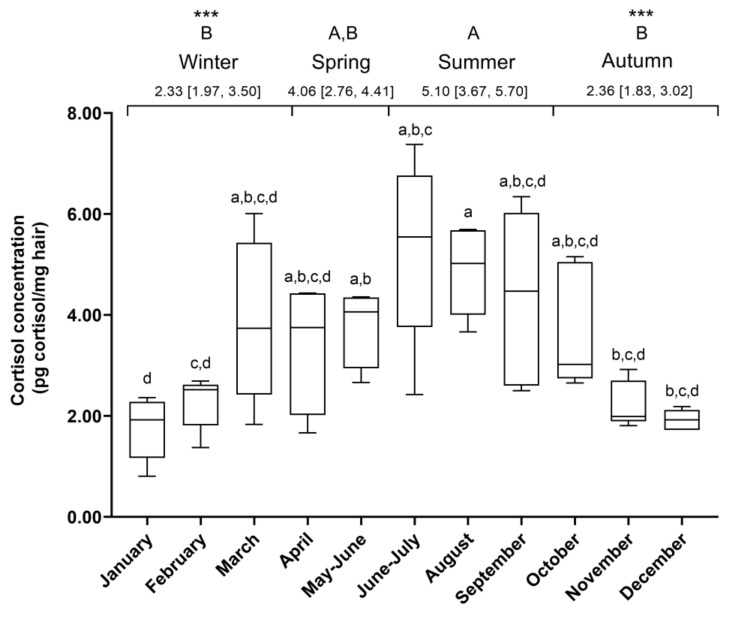
Fluctuation of hair cortisol concentrations over a year. Representation of hair cortisol concentrations (HCCs) (pg cortisol/mg hair) in horses (N = 5, n = 53) during a year. Different lowercase letters represent significant differences between months. Different uppercase letters represent significant differences between seasons (*** *p* < 0.001). Median (25% percentile, 75% percentile). N = number of horses per group; n = number of determinations of HCCs.

The HCC was higher in summer (5.10 (3.67, 5.70) pg cortisol/ mg hair) compared to autumn and winter (2.33 (1.97, 3.50) and 2.36 (1.83, 3.02) pg cortisol/mg hair, *p* = 0.0006 and *p* = 0.0002, respectively) (Figure 2). The HCC was similar in spring (4.06 (2.76, 4.41) pg cortisol/mg hair) and the rest of the seasons.

**Figure 3:**
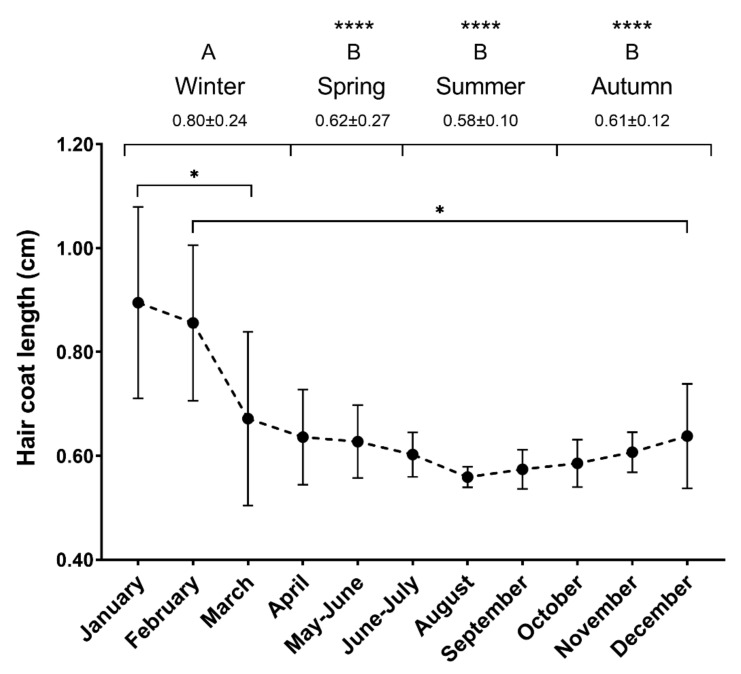
Hair growth rates over a year. Hair growth (cm) for each month and season in the horses included in the study (*n* = 5). Different uppercase letters represent significant differences between seasons. Different lowercase letters represent significant differences between months (* *p* < 0.05, **** *p* < 0.0001). Mean ±SD.

The mean hair growth rate on the ventral abdomen, left of mid-line and caudal to the sternum was 0.66 ± 0.11 cm/month (mean ±SD). Figure 3 shows the hair growth rate over the year of the study. The mixed-model analysis showed differences between January and March (0.90 ± 0.18 and 0.67 ± 0.17 cm, *p* = 0.0487), and between February and December (0.86 ± 0.15 and 0.64 ± 0.10 cm, *p* = 0.0372). Hair growth was faster in winter (0.80 ± 0.24 cm) compared to the rest of the seasons (*p* < 0.0001). Growth rates were similar in spring, autumn, and summer (0.62 ± 0.27, 0.61 ± 0.12 and 0.58 ± 0.10 cm, respectively, *p* > 0.05).

## 4. Discussion

To our knowledge, this is the first study to analyze the effect of a rest period and temporary relocation on HCCs in horses. The effects of seasonality, health status, age, hair growth, and coat color (bay vs. gray) were also analyzed.

Interestingly, the results of our study showed that the temporary relocation and rest period experienced by the police horses included in this study increased the HCCs, probably indicating increased HPA axis activity. However, the sample number was small and not representative of all sexes and breeds. Increasing the sample size and including more breeds and both sexes, will probably aid in increasing the statistical power of both studies. 

Animal welfare is affected by a complex interrelation of several factors [27]. Thus, our results suggest that an unexpected change in the multiple factors associated with a temporary relocation and resting period such as environment condition, housing system, habitual workload, nutrition, changes in staff and support, and social novelty may result in a wide range of stressors that increase the HPA axis activity in a long-term manner. 

Housing systems and management practices affect horses’ behavior [28]. Horses are usually housed in single stalls, but this type of housing restricts some natural behaviors and social interactions [28]. However, police horses spend most of their time training or patrolling with other horses, thus facilitating social interaction. During the relocation period, horses were subjected to solitary free exercise, which seems to be related to a higher degree of stress [28]. However, the external environment provided by the paddocks could ensure important behaviors and physiological needs [29]. Diet composition and changes in feeding frequency can produce fluctuations in horse hormone patterns, including cortisol [30,31]. Interestingly, cows relocated from their habitual environment, and nutrition conditions had higher HCCs [32]. In addition, staff turnover in a laboratory animal facility seems enough to produce an increase of HCCs in rabbits [33]. Taken together, all these potential stressors may have been reflected in the HCCs of the horses included in our study.

The circannual rhythm of cortisol concentrations has been well described previously in healthy and sick horses [34]. Higher plasma cortisol concentrations were found between summer and autumn season compared with the rest of the year [35,36]. Our study reported higher HCC in summer compared with autumn and winter. Nevertheless, the risk of a small sample size with not enough statistical power to identify differences in HCCs between seasons cannot be completely discarded. Slightly different range of cortisol concentrations were detected between our study and the one performed by Duran and colleagues [22], probably related to the different location of the hair sample. 

The body region and the type of hair could influence HCCs [9]. Our study showed a hair growth rate of 0.66 ± 0.11 cm/month on average in the ventral abdominal area. Different factors affect the seasonal coat shedding, including the season, genetic background, type of feeding, and housing systems [37,38,39]. This process aims to adjust the body temperature to the environmental conditions. A complete change of the summer coat to a winter coat should take place during late autumn [39]. From August until January, the hair growth rate increased, indicating a greater overall length during late autumn and early winter. In the same way, the highest hair growth rate was found in winter (0.80 ± 0.24 cm/season). Taken together, we observed a normal transition from a summer to a winter coat that usually starts in September in our latitude. 

In the present study, the health status did not have a significant influence on horse HCCs. The absence of an effect of the clinical episodes could be explained by the broad range of painless and painful disorders that were included in the health status assessment. In the study performed by Duran et al. [22], HCCs were higher one and two months after a very stressful acute procedure. In that study, horses were submitted to surgical intervention (castration) with complete anesthesia and analgesia protocols. However, lower HCCs have been reported in horses with severe squamous gastric disease when compared to horses without gastric ulcers [40]. Our study included a broad range of disorders that could mask a negative feedback produced by a high concentration of circulating glucocorticoids [41]. In other species such as cows, contradictory results concerning illness and HCCs have been reported [42,43].

No significant differences in HCCs between bay vs. gray hair color were detected in our study. In the same way, hair color had no significant influence on HCCs in bears and wolves [44,45]. However, some authors reported contradictory results regarding this issue in cows [10,25].

Within the range of ages of this study, we did not detect an effect on HCC. This result follows other reports that analyzed cortisol concentrations in other matrixes of the adult horse, such as salivary [46] and plasma cortisol [47]. However, higher HCCs have been found in new-born foals and 30–60-day-old foals compared with adult horses [21,22,23]. 

## 5. Conclusions

Our results suggest that HCC can be used to monitor the adaptation of horses to environmental and management variations, although the accurate nature of the relationship between HCC and welfare needs to be further investigated. Results of the present study could be applied to improve the management and environment of horses, which in turn could potentially improve the animals’ welfare. However, future studies with a larger sample size are needed to confirm these implications.
